# Immunomodulatory Role of Urolithin A on Metabolic Diseases

**DOI:** 10.3390/biomedicines9020192

**Published:** 2021-02-15

**Authors:** Ashley Mulcahy Toney, Darius Fox, Virginia Chaidez, Amanda E. Ramer-Tait, Soonkyu Chung

**Affiliations:** 1 Department of Nutrition and Health Sciences, University of Nebraska-Lincoln, Lincoln, NE 68583, USA; Df448@cornell.edu (D.F.); vchaidez2@unl.edu (V.C.); 2 Department of Food Science and Technology, University of Nebraska-Lincoln, Lincoln, NE 68583, USA; aramer-tait2@unl.edu; 3 Nebraska Food for Health Center, University of Nebraska-Lincoln, Lincoln, NE 68588, USA; 4 Department of Nutrition, University of Massachusetts-Amherst, Amherst, MA 01003, USA

**Keywords:** urolithin A, insulin resistance, mitochondria, inflammation, lipid metabolism, nutritional sciences, obesity, metabolic syndrome

## Abstract

Urolithin A (UroA) is a gut metabolite produced from ellagic acid-containing foods such as pomegranates, berries, and walnuts. UroA is of growing interest due to its therapeutic potential for various metabolic diseases based on immunomodulatory properties. Recent advances in UroA research suggest that UroA administration attenuates inflammation in various tissues, including the brain, adipose, heart, and liver tissues, leading to the potential delay or prevention of the onset of Alzheimer’s disease, type 2 diabetes mellitus, and non-alcoholic fatty liver disease. In this review, we focus on recent updates of the anti-inflammatory function of UroA and summarize the potential mechanisms by which UroA may help attenuate the onset of diseases in a tissue-specific manner. Therefore, this review aims to shed new insights into UroA as a potent anti-inflammatory molecule to prevent immunometabolic diseases, either by dietary intervention with ellagic acid-rich food or by UroA administration as a new pharmaceutical drug.

## 1. Introduction

Urolithin (Uro) production results from the bioconversion of ellagic acid through gut microbes. Ellagic acid (EA) is a polyphenol found in foods such as berries, pomegranates, and walnuts, and consumption of ellagic acid (EA)-rich foods yields ellagitannins (ETs), which hydrolyze in the stomach [1,2,3]. EA freely circulates in the stomach, and a proportion of EA reaches the small intestine and undergoes gut microbial conversion [4]. Due to low bioavailability of dietary polyphenols, the health benefits from pomegranate consumption are attributed to the released gut metabolites rather than the polyphenols per se [5].

The Uro family includes UroM-5, UroD, UroM-6, UroE, UroC, UroM-7, Iso-UroA, UroB, and UroA [6,7]. Recently, new “R” urolithins were discovered and classified as urolithin M7R, urolithin CR, and urolithin AR, thereby advancing knowledge regarding the various “metabotypes” which consist of producers collectively (UM-B) followed by non-producers labeled as metabotype 0 (UM-0) [6,8]. Uros possess a common 6H-dibenzo [b,d]-pyran-6-one nucleus and differ by hydroxyl groups [9]. Once absorbed, urolithins reach the liver and undergo phase 2 biotransformation, to yield various conjugated forms. For UroA, conjugated forms include UroA-glucuronide and UroA-aglycones [5]. Our review focuses on UroA because its conjugates are found at the highest concentrations in human plasma ranging from 0.024 to 35 μM [9]. This review aims to compile UroA’s anti-inflammatory role based on recent in vitro and in vivo studies. We specifically discuss UroA’s ability to combat inflammation within immune function, neurological disease, obesity, obesity-related metabolic syndrome, and cardiovascular disease. 

## 2. Current Status of Knowledge

### 2.1. Pharmacokinetics of UroA

**Figure 1:**
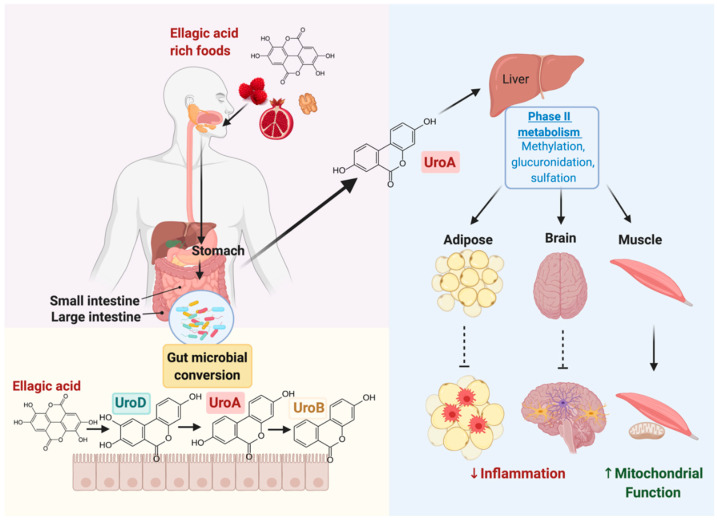
Ellagic-acid containing foods undergo gut microbial conversion to yield various forms of urolithins (Uro). Urolithin A (UroA) enters circulation and reaches the liver, to undergo phase II metabolism, to yield conjugated forms of UroA (such as methylation, glucuronidation, and sulfation). Conjugated UroA is able to reach peripheral tissues, including the adipose, brain, and muscle tissue, to prevent inflammation and increase mitochondrial function. Created with BioRender.com. UroA, urolithin A; UroB, urolithin B; UroD, urolithin D.

Urolithins are produced from ellagic-acid containing foods undergoing gut microbial conversion and vary in concentrations among individuals [10]. Once EA-containing foods reach the colon, the gut-derived metabolite UroA and its conjugates exert anti-inflammatory and anti-angiogenic activities, unlike UroA’s parental compound EA [11,12] (Figure 1). Moreover, methylated UroA inhibits miR-21 expression and its downstream targets to suppress prostate cancer progression [13]. In recent years, studies reported specific gut bacteria result in the production of UroA. As the story of UroA unfolds, researchers found specific gut metabotypes are associated with the release of specific urolithins, including UroA, iso-UroA, and UroB [14]. Individuals classified as metabolically healthy (i.e., without metabolic syndrome conditions) release higher concentrations of active UroA, therefore belonging to metabotype A [8,15]. Interestingly, those classified as metabolically unhealthy produce minimal urolithin A and release the less active forms of urolithin including iso-UroA and UroB, thus belonging to metabotype 0 and metabotype B, respectively [8,15]. Based on the correlations between metabotypes and metabolites, it suggests the gut microbial community may play a greater role in determining the production of active UroA. Of these microbes, *Gordonibacter urolithinfaciens* and *Gordonibacter pamelaeae* are involved in converting ellagic acid to UroA [16,17]. *Akkermansia muciniphila* levels are reported to be associated with UroA levels but can vary in UroA activity depending on inter-individual differences [18,19]. Based on this evidence, UroA production and activity may be associated with the gut microbial community and metabotype classification; however, more research is needed to provide stronger associations.

UroA possesses the ability to provide various health benefits to the host due to its specific chemical structure including acting as an estrogenic agonist identified through ligand docking, suggesting UroA’s potential ability to modulate endocrine activity [20]. Additionally, UroA is a natural microbiota-derived human-selective aryl hydrocarbon receptor ligand and aryl hydrocarbon receptors are expressed by numerous cells, including immune cells [21]. In connection with aryl hydrocarbon receptors, UroA induces the expression of various genes associated with phase 1 and phase 2 metabolism, also known as xenobiotic metabolism [22]. Through its ability to induce xenobiotic metabolism, UroA could potentially be a useful metabolite in attenuating disease through therapeutics.

As UroA research advances, the use of UroA therapeutics arises and highlights the importance of bioavailability and effectiveness of this metabolite. UroA has been demonstrated to reach peripheral tissues both through oral administration and injections; however, several studies classify UroA’s actions as dependent on its conjugation with glucuronide, aglycone, or sulfation [9,23]. This notion was recently challenged, as researchers have demonstrated that free UroA reaches peripheral tissues, thereby suggesting deconjugation from UroA glucuronide [23]. Specifically, in inflammatory conditions, conjugated UroA converts into free UroA to exert its health benefits in tissues other than the intestine [23] (Figure 1). Therefore, we focus on UroA’s ability to modulate inflammation in various cell types and tissues, including studies reporting both free UroA and its conjugated forms since more research regarding UroA’s pharmacokinetics and attenuation of disease is warranted.

### 2.2. Immunomodulatory Function of UroA

Innate immune system activation is crucial for acute danger signals in response to injury or trauma; however, under chronic activation, this can lead to cell cytotoxicity. One mechanism of the innate immune system utilizes myeloperoxidase (MPO) which is expressed in neutrophils [24]. Upon respiratory burst of neutrophils, MPO mediates the generation of H_2_O_2_ to kill bacteria; however, under circumstances of chronic activation, inflammation and tissue damage ensue [24,25]. Saha et al. investigated whether UroA inhibits the generation of toxic H_2_O_2_ and inflammation. Using a phorbol myristate acetate (PMA)-induced ear edema model in mice, researchers observed that 40 mg/kg of UroA before the onset of edema was enough to inhibit heme peroxidases by inactivating the peroxidase-catalyzed reaction and subsequent unnecessary generation of reactive oxygen species (ROS) in neutrophils [26]. Furthermore, in human neutrophils, UroA decreased ROS production in response to lipopolysaccharide (LPS) stimulation, suggesting anti-oxidative properties and potential in modulating neutrophil function [27]. In all, UroA exerts protection against oxidative stress and pro-inflammatory stimuli.

Similar to the actions in neutrophils, UroA inhibited the generation of LPS-induced ROS in murine macrophages and peritoneal macrophages [28]. UroA suppressed pro-inflammatory cytokines, including tumor necrosis factor alpha (TNFα) and interleukin 6 (IL6), decreased nitrite and inducible nitric oxide synthase (iNOS) production, by inhibiting the activation of NFκB signaling pathways [28]. Consistently, studies reported UroA’s ability to suppress the protein kinase B (Akt)/mitogen-activated protein kinase (MAPK) signaling pathways by either modulating the nuclear translocation of NFκB, inhibiting pro-inflammatory cytokine production, or reducing oxidative stress in various immune cells [28,29,30,31,32,33]. In supporting this notion, multiple research studies confirm UroA as a potent mediator to reduce inflammation associated with innate immunity and ROS production, as found in Table 1.

### 2.3. Modulation of Autophagy by UroA

Beyond inhibition of NFκB activation, UroA enhances autophagy under inflammatory conditions to enhance cell viability. Using J774.1 murine macrophage stimulated with LPS, Boakye et al. demonstrated that UroA suppressed the pro-inflammatory M1 macrophage polarization and subsequently dampened pro-inflammatory cytokine secretion [34]. Furthermore, UroA inhibited M1 macrophage polarization by increasing autophagic flux, which is necessary for impeding nuclear translocation for the activation of Akt/mTOR signaling pathways [34]. To further support evidence of UroA’s mechanism of action as autophagy, Ryu et al. illustrated UroA’s function in promoting mitophagy in *Caenorhabditis elegans* (*C. elegans*) nematode and rodents. UroA administration (1) extended survival and lifespan in *C. elegans*, (2) improved mitochondrial function to suppress aging *C. elegans* and rodents, and (3) promoted the survival and increase muscle function via mitophagy in rodents [35]. Likewise, UroA increased skeletal muscle function by increasing ATP and NAD+ levels through Sirtuin 1 (Sirt1) and the peroxisome proliferator-activated receptor gamma coactivator 1-alpha (Pgc1α) upregulation, thereby connecting UroA’s enhancement of mitochondrial function [36]. To summarize, current literature supports the notion that UroA acts on inflammation and aging by promoting mitochondrial function, specifically mechanisms dependent on mitophagy (Table 1).

## 3. Impact of UroA on Immunometabolic Diseases

### 3.1. Neuroinflammation and Neurodegenerative Diseases

In the past year, the focus on UroA’s ability to mitigate neuroinflammation has increased, with increasing evidence displaying UroA’s ability to cross the blood–brain barrier (BBB) characterized in silico and in vivo [37,38,39]. In a neuroinflammatory environment, microglia polarize toward a pro-inflammatory state and release a stream of pro-inflammatory cytokines including IL6, TNFα, and nitric oxide (NO) [40]. Upon secretion, these cytokines generate a pro-inflammatory environment that allows for protein dysfunction, such as seen in Alzheimer’s disease (AD), and rampant apoptosis [41].

As UroA exerted anti-inflammatory properties in macrophages, UroA inhibited neuroinflammation in aged transgenic R1.40 mouse hippocampus, human neuroblastoma cells, and BV2 mouse microglial cells [42]. UroA decreased total NO concentration and pro-inflammatory cytokines IL6 and TNFα, as well as increasing cell viability by inhibiting apoptosis [42]. Therefore, UroA is shown to prevent overactivation of microglia by preventing neuroinflammation and decreasing apoptotic pathways to enhance neuron cell viability. Xu et al. also finds that UroA is able to prevent neuroinflammation in BV2 mouse microglial cells upon stimulation by LPS. Comparably, Xu et al. find UroA reduces NO production, inhibits pro-inflammatory cytokines; furthermore, UroA suppressed the NFκB signaling pathway, MAPK signaling pathway, and the PI3K/Akt pathways [43], similar to UroA’s influence on immune cells outlined above. Low concentrations of UroA suppressed apoptosis and ROS production through regulation of the p38-MAPK [44] and increasing AMPKα [45], suggesting UroA as a promising metabolite in mediating the detrimental effects of oxidative stress consistent in neurodegenerative diseases. Furthermore, UroA suppresses oxidative stress and confers neuroprotection in Neuro-2A cells by acting as a radical scavenger and enzyme inhibitor of oxidases to suppress inflammation [46]. Therefore, UroA consistently exerts anti-inflammatory actions inhibiting NFκB signaling pathways and mediating oxidative stress in neuronal cells.

Given AD pathology is associated with reactive gliosis, inflammatory environments within the brain produce proinflammatory cytokines, increase amyloid precursor protein (APP) expression, and promote Amyloid β (Aβ) deposits in the brain [47]. In vivo, UroA reduced neuroinflammation in APP/PS1 transgenic female mice exhibiting an AD pathophysiology [48]. Specifically, UroA has been shown to prevent learning and memory deficits; deter cell death; and alleviate plaque production, Aβ levels, and reactive gliosis [48]. One possible mechanism for UroA’s ability to reduce Aβ levels would be autophagy, depending on mitochondrial function. In microglial and neuronal cells, low concentrations of UroA is potent in inhibiting NFkB acetylation and Aβ production through activation of autophagy and Sirt-1 [49]. Furthermore, another aging mouse model reported UroA activated miR-34-a to induce SIRT1/mTOR signaling [50]. Using a d-galactose aging mouse model, an oral administration of UroA prevented a brain-related aging disease by inducing hippocampal autophagy, as well as suppressing inflammation [50]. Since UroA research suggests macroautophagy or specifically mitophagy may be a targeted benefit, Ahsan et al. sought to understand UroA’s mechanism on mitophagy versus autophagy toward suppressing inflammation. In mice and neuroblastoma cells, UroA reduced cell injury and modulated ER stress through upregulation of autophagy [51]. In contrast to previous studies, Ahsan et al. reported that UroA does not activate mitophagy but rather enhances general macro-autophagy to confer neuroprotection [51]. It is still controversial whether UroA exerts protection through general autophagy, mitophagy, or autophagy in conjunction with mitochondrial function. Nonetheless, UroA consistently protects against neuroinflammation, as summarized in Table 2.

### 3.2. Cardiovascular Disease

In response to toll-like receptor 4 (TLR4) stimulation, Akt reaches the plasma membrane of the cell and allows for secretion of various cytokines [66]. Interestingly, depending on the activation signal, Akt can result in either pro-inflammatory or anti-inflammatory responses [66], modulating M1/M2 polarization [67]. In endothelial cells, PI3K/Akt is important for vascularization and is responsible for anabolic processes [52]. However, over-activation of Akt plays a role in the pathogenesis of diabetic vascular activation [52]. UroA subdued Akt signaling pathways in human endothelial cells for management of type 2 diabetes mellitus, possibly due to its unique chemical structure as other urolithins failed to exhibit similar actions [52]. UroA regulated Akt/β-catenin signaling pathways in vascular smooth muscle cells by suppressing the phosphorylation of Akt at the residue Threonine 308, as well as modulating β-catenin pathways, to reduce ROS and inflammation [53]. In vivo, the cardioprotective effects of UroA were investigated in a mouse model of myocardial reperfusion injury, defined as extensive tissue damage occurring after blood supply is restored post-ischemic injury or period of hypoxia [54]. UroA reduced ROS production by regulating the PI3K/Akt pathway and enhancing antioxidant activities in mice and cardiomyocytes [54]. In contrast to the previous two studies, UroA attenuated myocardial injury by activating the PI3K/Akt signaling to reduce apoptosis [54]. In summary, regarding vasculature and endothelial cells, UroA regulates the PI3K/Akt pathway and downstream targets to limit inflammation after cardiovascular injury, however more research is needed to confirm the clinical benefits of UroA against ischemia or hypoxia.

Inflammation and endothelial dysfunction exacerbate atherosclerosis [68]. Moreover, oxidized low-density lipoprotein (oxLDL) is a hallmark of atherosclerotic pathophysiology. With an increase of oxLDL, endothelial dysfunction occurs due to increases in inflammation and impairment of NO which is vital for endothelial function [69]. Han et al. investigated UroA’s effect on impairing oxLDL-induced endothelial dysfunction and inflammation in human artery endothelial cells treated with oxLDL. Low concentrations of UroA were shown to increase NO production due to improvement of endothelial nitric oxide synthase (eNOS), decrease monocyte adhesion factors such as ICAM-1, and decrease ERK-mediated inflammation along with IL6 and TNFα [55]. Therefore, UroA confers protection against endothelial dysfunction caused by oxLDL through reduction of inflammation and restoration of NO production. Atherosclerosis is multifaceted and oxLDL is but one factor for inducing its inflammatory pathophysiology. As atherosclerosis progresses, cardiac ischemia may occur due to scavenger receptor-class B type 1 (SR-B1) inhibition as SR-B1 mediates cholesterol transport of high-density lipoprotein (HDL) to the liver and reduces atherosclerotic plaque formation [56]. Cui et al. further examines whether UroA is able to attenuate atherosclerotic lesions in rats fed a high-cholesterol diet and subjected to a balloon injury of the aorta. UroA reversed inflammatory lipid levels, decreased levels of angiotensin II, and decreased foam cell development through activation of the Nrf2 pathway and inhibition of p-ERK [56]. Additionally, in macrophage foam cells, UroA suppressed mIR-33-a by altering ERk/SREBP1/AMPKα signaling pathways to regulate cholesterol efflux to reduce intracellular cholesterol load [57]. Lastly, UroA improved hemodynamics and decreased inflammation of CX3CL1 in a rat model of diabetic cardiomyopathy [58]. In all, UroA confers protection against atherosclerosis by restoring NO production, mediating cholesterol transport, and regulating ERK signaling pathways (Table 2).

### 3.3. Obesity, Type 2 Diabetes, and Metabolic Syndrome

A precursor to cardiovascular disease (CVD) is obesity and associated metabolic syndrome. Obesity is detrimental to health and is associated with the induction of metabolic syndrome. Metabolic syndrome is classified as high waist circumference, elevated triglycerides, reduction of high-density lipoprotein cholesterol, high blood pressure, and high fasting blood glucose [70]. In individuals with three classical metabolic syndrome risk factors fed 30 g of raw nuts for 12 weeks, UroA glucuronide was found to be at the highest concentration in plasma [71]. Moreover, UroA glucuronide in plasma was inversely correlated with abdominal adiposity defined by waist circumference [71]. Therefore, in a human population, the UroA conjugate, UroA glucuronide, reversed metabolic syndrome as shown in abdominal adiposity.

Mechanistically, Kang et al. reported UroA (in addition to UroC and UroD) attenuated triglyceride accumulation in both human adipocytes and hepatocytes by decreasing lipogenic gene expression [59]. UroA increased fatty acid oxidation through AMPK-dependent mechanisms to decrease triglyceride accumulation [59]. In 3T3-L1 murine pre-adipocytes, UroA decreased triglyceride content and abolished the expression of PPARγ, Glut4, and FABP4 in pre-adipocytes [60] and dampened lipogenesis without impairing adipogenesis [61]. Recent studies suggest UroA protects against high-fat-diet-induced obesity and related insulin resistance. With gavaging UroA, mice were protected from high fat diet-induced obesity and insulin resistance through augmentation of thermogenesis stimulated by thyroid hormone signaling to induce browning [62]. Toney et al. also demonstrated UroA’s beneficial effects using an in vivo model of C57BL/6 mice fed a high-fat diet and injected intraperitoneally with UroA daily for 90 days [63]. UroA improved insulin sensitivity, decreased hepatic triglyceride accumulation and inflammation, and decreased adipose tissue macrophages and hypertrophy by inhibiting M1 macrophage polarization while promoting M2 macrophage polarization and mitochondrial function [63]. In supporting this study, daily intraperitoneal injection of UroA protected rats against high fat diet-induced obesity by attenuating oxidative stress, decreasing lipogenesis and providing antioxidative protection [64]. On the contrary, in DBA2/J mice fed a high-fat/high-sugar diet, UroA supplementation in the diet (0.1%) decreased fasting blood glucose, but failed to improve insulin sensitivity; however, compared to its EA precursor, UroA supplemented diet increased adiponectin and improved mitochondrial function in the liver and skeletal muscle, consistent with UroA beneficial mechanisms in multiple disease states [65]. This difference between studies could be due to supplementation of UroA (diet versus injection/gavage), diet intensity (high fat versus high fat/high sugar)/duration or experimental animal model. More studies are summarized in Table 2. From the summary of these studies, UroA attenuated obesity and metabolic syndrome seen both in vivo and in vitro. For future study direction, more research is warranted to define the exact mechanisms involving the gut microbiome to elicit these health benefits.

### 3.4. Nephrotoxicity

For chemotherapy patients using cisplatin, nephrotoxicity can occur as a side effect to this drug or in combination with other chemotherapy drugs. Several studies have demonstrated the effectiveness of intraperitoneal injection or orally administered UroA in attenuating nephrotoxicity in mice. UroA treatment prevents cisplatin-induced nephrotoxicity in Sprague-Dawley rats by decreasing pro-inflammatory cytokines while increasing IL-10; moreover, UroA decreased NFκB activation and inhibited proapoptotic pathways [72]. In another study, UroA provided protection against cisplatin-induced nephrotoxicity due to its anti-oxidant and anti-inflammatory properties [73]. Only using 100 mg/kg per day intraperitoneal injection, researchers found that UroA modulated oxidative stress and inflammation caused by cisplatin by reducing pro-inflammatory cytokines, infiltrating leukocytes, and reactive oxygen species production [73]. Within the kidney, UroA induced autophagy, to protect against ischemia-reperfusion injury (IRI) and kidney dysfunction, by decreasing TNFα and IL-1β secretion [74] (Table 3).

## 4. Development of UroA as a Therapeutic

As summarized in Table 1, Table 2 and Table 3, UroA is a promising biomarker for gut health and gut composition, based on its health-benefitting effects driven by the gut microbiota and host metabolism [75]. Moreover, UroA can potentially be used as a cardiometabolic biomarker in humans [76]. Given that UroA’s beneficial effects on gut health has been established in the past decade, using UroA as a biomarker may correlate health status among gut, metabolic, and neurological samples, for future prevention purposes. However, bioavailability of either ellagic acid or its gut metabolite UroA may pose a problem in its potency, as polyphenol bioavailability is limited, and emerging research involving metabotypes suggest the host’s microbiota plays a role in producing UroA.

Recently, advances have been made to increase the availability of UroA in similar synthetic forms. Singh et al. developed a synthetic analogue to UroA, named UAS03, which has been shown to resist stomach acid degradation, increase gut health through enhanced barrier function, and reduce inflammation throughout the host [77]. Essentially, UAS03 is a generated non-hydrolysable cyclic ether derivative of the naturally occurring gut metabolite UroA; however, it remains stable [77]. Therefore, focusing efforts on stably producing the effects of UroA through pharmaceutical advances may provide future therapeutics for various diseases.

Moreover, as UroA continues to be tested in a human population, its health benefits are being better understood. Recently, Andreux et al. found that UroA packaged in encapsulated soft gels for oral administration in an elderly population is safe and mimics an exercise response in muscle, due to its mitochondrial activities [78]. In this healthy population, they did find that conjugated UroA metabolites were found at higher concentrations, thereby suggesting under a healthy participant UroA undergoes phase-two conjugation [78]. Lastly, they found that UroA administration increased fatty acid oxidation and mitochondrial function in human skeletal muscle despite a sedentary trial [78]. Increasing evidence suggesting UroA as a potential exercise mimetic is warranted and may provide a clue for combating obesity and related disease.

Despite one method of orally ingesting UroA in an encapsulated form, other research should focus on developing the targeted delivery of the parent compound UroA, instead of its conjugated forms, to various target tissues and organs using nanotechnology. One suggestion is to research modes of delivery using nanoparticles encapsulating UroA. As of now, one study has focused on developing a biodegradable nanoparticle to package UroA to increase bioavailability; this has been tested only in mice, but it has shown promising results in protecting against acute kidney injury, improving survival rate, and mitigating oxidative stress [79]. In the case of pomegranate bioactive compounds, researchers found that pomegranate nanoprototypes are able to decrease proliferation of breast cancer cells by increasing bioavailability [80]. However, these findings for this pomegranate nanoprototype and UroA biodegradable nanoparticle need to be further tested in humans, for translational implications.

Lastly, another method of increasing UroA efficacy is through fecal microbiota transplants (FMTs), which have gained traction in the clinical field. FMT is mainly used for treating *Clostridium difficile* infection which is antibiotic resistant [81]. FMT uses a healthy donor’s stool to reach the recipient’s intestine, where the healthy microbial community is able to colonize and enact changes in the microbiome [81]. In terms of UroA delivery, metabotype 0 individuals who do not possess the microbes to convert ellagic acid to UroA may be eligible for FMT from a metabotype A donor. In doing so, this can potentially allow metabotype A microbes to colonize the recipient’s microbiome and allow for UroA conversion and functionality. However, this warrants further research, and donor screening practices will need specific criteria in order to prevent transfer of infection or other adverse reactions.

## 5. Conclusions

Chronic inflammation induces various metabolic pathologies in the brain, heart, adipose, and kidney. In this review, we focused on the current developments in UroA research concerning immune modifications, neuroinflammation, metabolic and cardiovascular diseases, and nephrotoxicity. Moreover, we proposed UroA’s mechanisms of action in attenuating these diseases by targeting mitochondrial function, induction of autophagy, mitigation of MAPK/NFkB/Akt signaling, and reduction of ROS and pro-inflammatory cytokines in multiple tissues. Currently, UroA is being investigated as a potential pharmacological disease-modifying oral supplement due to its various biological effects in promoting mitophagy and limiting inflammation. Moreover, research focused on discerning the different metabotypes, its relation to urolithin production, and gut microbes associated with these metabotypes will yield answers toward the conversion of ellagic acid into various bioavailable urolithins for individuals with unique microbiomes and health conditions. Therefore, future research focused on elucidating the conversion of EA into urolithins and the safe efficacy of using UroA as a drug can yield promising therapeutics.

## Figures and Tables

**Table 1:** Immunomodulatory function of UroA.

Category	Test Model	Disease Type/Treatment	Dose (UroA)	Metabolic Response	Ref.
Immune	C57BL/6 mice	Edema	40 mg/kg/BW orally	↓ MPO activity and LPO activity with iron chelation ↓ ear edema weight (mg)	[26]
	BMDM		10 μM (BMDM)		
Immune	Ex vivo human neutrophils	LPS inflammation	20 μM	↓ IL-18 production (%), MMP-9 production (%), MPO release (%) ↓ ROS release (%) ↓ Superoxide anion production activity (%) and ↑ uric acid production activity (%)	[27]
Immune	RAW265 murine macrophages and peritoneal macrophages	LPS inflammation	2–40 μM	↓ TNFα, IL-6, nitrite, iNOS ↓ DNA binding response to LPS, LPS induced translocation of p65 ↑ IkBα ↓ AP-1 DNA binding activity, c-JUN and p-c-JUN ↓ p-Akt, p-JNK, p-p38 ↓ NOX (ROS)	[28]
Immune	Human osteoarthritis chondrocytes	Osteoarthritis	3–30 μM	↓ Il-1β, iNOS, Cox-2, NO generation, PG32 ↓ IkBα degradation and translocation of P65 to nucleus ↓ PI3K/Akt signaling pathway ↓ p-PI3K and p-AKT-positive chondrocytes in mouse ↓ p65-positive nuclei in UA mouse chondrocytes; milder narrowing of joint space compared to OA group	[29]
	DMM mouse model		20 mg/kg/day intragastric administration		
Immune	Rat chondrocytes	Osteoarthritis	1–15 μM	↓ MMP13, MMP3, iNOS, Cox2, ADAMST4, MMP9 ↑ Col2a1 ↑ Collagen II, Aggrecan, Sox9 ↓ p65, p-ERK1/2, p-JNK, p-P38	[30]
Immune	U937 cell THP-1	LPS inflammation	1.5, 30 μM	↓ Tnfα ↓ NFκB signaling, p50 and p60 subunits	[31]
Immune	RAW 264.7 murine macrophages	LPS inflammation	2–40 μM	↓ NO production, nitrite, iNOS ↓ NF-κB p65 nuclear translocation ↓ binding to NFκB p50 binding ↓ IL-1β, TNFα, IL-6	[32]
Immune	THP-1, RAW 264.7 PBMCs, neutrophils	LPS inflammation	40 μM	↓ Tnfα ↑ TGFβ1 ↓ p65, ↑ p-ERK ↓ iNOS	[33]
Autophagy	J774.1 macrophage, HEK 293 CHO-ARE-LUC reporter	LPS inflammation	20–40 μM	↓ NO release, ROS, pro-IL-1β ↑ LC3 II activation ↓ p-AKT (T308), pTSC2 (T1462), p-p70S6k ↓ iNOS, pro-IL1β, COX2 ↓ nuclear p65	[34]
Autophagy	*C. elegans*	HF-diet induced obesity (60% kcal from fat)	10–50 μM (*C. elegans* and C2C12 myoblasts) 25 mg/kg/day in food pellets (rat studies) 50 mg/kg/day in food pellets (mouse studies)	↑ survival, pharyngeal pumping per min, mobility, muscle fiber organization ↑ cco-1, phi-37, mev-1, sdha-1, and O2 consumption by day 8 in *C. elegans* ↑ formation GFP-LGG-1-positive punctae, mitochondrial content in muscle and intestine by day 5 *C. elegans*	[35]
	C2C12 myoblasts			↑ LC3-II/LC3-I ratio in intestinal cells and ubiquitination and p62/SQSTM in mitochondrial fraction of intestinal cells, phospho-AMPKα, LC3II in C2C12 myoblasts, formation of endogenous autophagosomes	
	Sprague-Dawley and Wistar rats				
	C57Bl/6 mice			↓ basal O2 consumption, ATP content ↑ grip strength, running distance (km), p-AMPKα, LC3II, ↓ p-62, ↑ pik3c3, park2, ub/sdha, ub/vdac in rodent models	
Autophagy	C57Bl/6 mice	Muscle angiogenesis	10 mg/kg/BW gavage	↑ VEGFA, CDH5 ↑ SIRT1, PGC-1a ↑ ATP and NAD+ levels	[36]
	C2C12 myoblasts		10 μg/mL		

UroA administration is specified for rodent and human studies; for cell studies, dose is in indicated in μM. Akt, protein kinase B; AMPKa, AMP-activated protein kinase alpha; BMDM, bone marrow-derived macrophages; BW, body weight; FMT, fecal microbiota transplantation; HF, high fat; IL1b, interleukin 1-beta; IL6, interleukin 6; IL10, interleukin 10; LPO, lactoperoxidase; iNOS, inducible nitric oxide synthase; LPS, lipopolysaccharide; MPO, myeloperoxidase; NAD+, nicotinamide adenine dinucleotide; NF-κB, nuclear factor-kappa B; NO, nitric oxide; ROS, reactive oxygen species; Sirt-1, sirtuin 1; TNFa, tumor necrosis factor.

**Table 2:** Metabolic function of UroA.

Category	Test Model	Disease Type/Treatment	Dose (UroA)	Metabolic Response	Ref.
Neuro	R1.40 mouse hippocampal tissue SH-SY5Y neuroblastoma BV-2 mouse microglia	LPS for AD model	10 μM	↓ total NO ↓ IL-6 and TNFα ↑ cell viability ↓ caspase 9, caspase 3/7 release	[42]
Neuro	BV-2 mouse microglia	LPS inflammation	3- 30μM	↓ NO, TNFα, IL-6, iNOS, COX2, IL-1β ↓ supernatant TNFα, IL-6, IL-1β ↑ IkBα ↓ nuclear p65, p-p65, p-IkBα ↓ p-ERK 1/2, p-p38, p-Akt	[43]
Neuro	SK-N-MC human neuroblastoma	Neuroblastoma	1–5 μM	↑ cell viability ↓ intracellular ROS levels ↓ Bax/Bcl2 ratio ↓ CytC, cleaved caspase9, cleaved caspase3, cleaved PARP ↓ p-p38/p38	[44]
Neuro	MCAO SPF mice	Cerebral ischemia	1.5–2 mg/kg/BW in food pellets	↑ mNSS, ↓ spatial memory deficits ↑ BrdU+ cells, DCX+ cells in dentate gyri ↓ TUNEL-positive cells ↓ Bax, Caspase 3, and ↑ Bcl2 ↓ Il-6, Iba1 cells in hippocampus, Tnfα, Il-1β, GFAP+ cells in hippocampus ↑ p-AMPKα, p-Ikbα ↓ p-Akt/Akt, p-p65 NFκB/p65NFκB, p-ERk1/2, p-JNK, p-p38	[45]
Neuro	Neuro-2a cells	H2O2 treatment	0.5–20 μM	↓ ROS ↓ TBARs ↑ catalase activity, SOD, GR activity, GPx activity ↑ Prdx1, Prdx3	[46]
Neuro	APP/PS1 transgenic mice	Neuroinflammation	300 mg/kg BW UroA orally	↑ learning and memory deficits ↓ cell death ↑ hippocampal neurogenesis ↓ Aβ plaque number ↓ Il-1β, Il-5, Tnfα ↑AMPKα ↓ p-p65 NFκb, p38 MAPK, Bace1, APP	[48]
Neuro	BV-2 mouse microglia, HEK293, ReNcell VM cells	LPS inflammation	2.5–10 μM	↓ nitrite, TNFα, IL-6 ↓ phospho-p65, acetyl-p65 ↑ SIRT1 ↑ autophagy ↓ LDH release (p < 0.001) ↓ Aβ production	[49]
Neuro	D-galactose mice	Brain aging	150 mg/kg/day subcutaneous injection	↑ miR-34a mediated SIRT1/mTOR signaling pathways	[50]
	PC12 rat cells		50, 30, 10 μg/mL	Inhibit apoptosis by ↑ autophagy	
Neuro	C57Bl/6 mice	MCAO focal cerebral ischemia	2.5 or 5 mg/kg/BW intraperitoneal injection	↓ LDH (U/L) in N2a and primary neuronal cells ↑ autophagy LC3 puncta in mCherry-LC3 transfected N2a cells and primary neurons ↑ LC3 II protein (N2a and primary neurons) ↓ p62 in mice brains ↓ ATF6, CHOP mRNA in N2a cells and brains ↓ infarct volume (%) and Neurological Deficit Score in mice	[51]
	Neuro-2a neuroblastoma		3–30 μM		
CVD	Ea.hy926 HUVEC endothelial cell	Diabetic Vascular disease	10 μM	↓ p-Akt Ser473/Akt total	[52]
CVD	A7r5 VSMC	Vascular smooth muscle dysfunction	5–40 μM	↓ p-Akt Thr308, total B-catenin, c-myc, cyclin D1	[53]
CVD	C57Bl/6 mice	Myocardial reperfusion injury	1 mg/kg/BW UroA intraperitoneal injection	↓ INF/AAR and INF/LV, TUNEL-positive cells, ↑ Ejection Fraction, Fractional Shortening, and ↓ CK, LDH ↓ ROS, MDA and ↑ SOD ↑ Cell viability ↑ p-PI3K/total PI3K, p-Akt/total AKT, Bcl-2/Bax, and ↓ cleaved caspase 3	[54]
	Neonatal rat cardiomyocytes		10 μM		
CVD	HAECs	oxLDL	0.5–5 μM	↓ LDH concentration ↑ NO and eNOS ↓ ICAM-1 and MCP-1 mRNA ↓ IL-6, ET-1 and ↑ PPARγ ↓ TNFα ↓ p-ERK/ERK, IL-6, ↑ PPARγ	[55]
CVD	Wistar rats	Atherosclerosis	3 mg/kg/BW orally	↓ serum TC, TG, LDL ↓ p-Erk and AT1, ↑ SR-B1 aortic tissue ↓ chemotaxis by RANTES and MCP-1 ↑ HO-1, NQO-1, Nrf2 activity ↓ foam cells	[56]
CVD	RAW 265.7	Atherosclerosis	5–20 μM	↓ intracellular cholesterol ↑ extracellular cholesterol ↓ p-ERK, SREBP1 ↑ p-AMPKα ↓ miR-33a ↑ ABCA1 and ABCG1	[57]
CVD	Wistar rats	Diabetic cardiac dysfunction	2.5 mg/kg/day intraperitoneal injection	↓ CX3CL1	[58]
Obesity/MetS	hASC	Triglyceride accumulation	30 μM	↓ lipogenesis, Fas, aP2, PPARγ, CEBPα, ATGL, SCD-1 ↑ p-AMPKα ↑ FA oxidation	[59]
Obesity/MetS	3T3-L1 preadipocytes	Triglyceride accumulation	10 and 50μM	↓ Triglycerides ↓ PPARγ, Glut4, FAB4	[60]
Obesity/MetS	3T3-L1 preadipocytes	Lipogenesis	25 μM	↓ intracellular triglyceride ↓ PREF-1 ↓ Glut4, Adiponectin, Leptin ↓ Tnfα, iNOS	[61]
Obesity/MetS	C57BL/6 mice	High-fat-diet-induced obesity	30 mg/kg/day gavage	↓ BW gain, fat mass and plasma glucose ↑ glucose uptake ↑ EE ↑ thermogenesis ↑ T3 in BAT and iWAT, ↓ T4 in iWAT	[62]
Obesity/MetS	C57BL/6 mice	High-fat-diet-induced obesity	20 μg/mouse intraperitoneal injection	↓ blood total cholesterol, LDL ↑ plasma adiponectin ↓ liver triglycerides, ↓ Huh7 lipogenesis ↓ epidydimal adipose hypertrophy ↑ mitochondrial biogenesis ↓ M1 macrophage markers Cd11c & Tnfα IL-6 & Mcp1 & IL1β ↑ M2 macrophage markers Chi3l3 (Ym1) & Mgl2 in peritoneal macrophages	[63]
	Huh7BMDM		30 μM		
Obesity/MetS	Wistar rats	Obesity	2.5 mg/kg/BWintraperitoneal injection	↓ body weight ↓ TBARS, ↑ SOD ↓ hepatic lipids, hepatic TG, hepatic chol, fecal lipid load ↓ LXRα, SREBP1c, P-ERK, IRE1a ↑ PPARα	[64]
Obesity/MetS	DBA2/J mice	HF/HS diet induced IR and Obesity	0.1% supplemented in diet	↓ fasting glucose concentration ↓ serum FFA, ↑ serum adiponectin, ↑ Pink1, Prkn, Mfn2 in liver; Mfn2 in skeletal muscle	[65]

UroA administration is specified for rodent and human studies; for cell studies, dose is in indicated in μM. AD, Alzheimer’s disease; APP, amyloid precursor protein; eNOS, endothelial nitric oxide synthase; ET, ellagitannins; FABP4, fatty acid-binding protein 4; FFA, free fatty acids; GLUT4, glucose transporter type 4; GR, glutathione reductase; GPx, glutathione peroxidase; HS, high sugar; LDL, low-density lipoprotein; MAPK, mitogen-activated protein kinase; MDA, malondialdehyde; oxLDL, oxidized low-density lipoprotein; SOD, superoxide dismutase; TBARs, thiobarbituric acid-reactive products; TC, total cholesterol; TG, triglycerides.

**Table 3:** UroA in kidney disease.

Category	Test model	Disease Type/Treatment	Dose (UroA)	Metabolic Response	Ref.
Kidney	Sprague-Dawley rats	Cisplatin	50 mg/kg BW orally	↓ plasma creatinine levels ↑ protection against epithelial necrosis ↓ TIM-1, NFκB expression, Iba1, TNFα, IL-6, IFNγ, IL-1α, IL-1β, IL-13, IL-17A, IL-2 ↑ IL-10 and NOS-3 ↓ tubular cell apoptosis (number of TUNEL positive cells)	[72]
Kidney	C57Bl/6 mice	Cisplatin	100 mg/kg BW intraperitoneal injection	↓ NGAL, BUN, Creatinine, Urinary KIM-1 ↓ tubular damage score ↓ TNFα, IL-23, IL-18, MIP2 ↓ CD11b positive cells in kidney ↓ HNE Protein Adducts Protein Nitration, Caspase 3 activity, DNA fragmentation ↑ GSH, GSH/GSSG ratio, ↑GSSG ↓ NOX2 ↑ Glutathione Peroxidase Activity and SOD activity	[73]
Kidney	C57BL/6 mice	Ischemia reperfusion injury	50 mg/kg BW (not specified)	↓ BUN, NGAL, Creatinine, KIM-1 ↓ TNFα, IL-1β, MIP-1α, MIP2	[74]

UroA administration is specified for rodent and human studies; for cell studies, dose is in indicated in μM. BUN, blood urea nitrogen; GSH, glutathione; GSSG, glutathione disulfide; NGAL, neutrophil gelatinase-associated lipocalin;

## Abbreviations

AD	Alzheimer’s disease
Akt	protein kinase B
AMPKa	AMP-activated protein kinase alpha
Ab	amyloid beta
APP	amyloid precursor protein
BBB	blood–brain barrier
*C. elegans*	*Caenorhabditis elegans*
CRP	C-reactive protein
EA	ellagic acid
ET	ellagitannins
eNOS	endothelial nitric oxide synthase
FABP4	fatty acid-binding protein 4
FMT	fecal microbiota transplantation
GLUT4	glucose transporter type 4
HDL	high-density lipoprotein
IL1b	interleukin 1-beta
IL6	interleukin 6
IL10	interleukin 10
iNOS	inducible nitric oxide synthase
LPS	lipopolysaccharide
MAPK	mitogen-activated protein kinase
MPO	myeloperoxidase
NF-κB	nuclear factor-kappa B
NO	nitric oxide
oxLDL	oxidized low-density lipoprotein
PMA	phorbol myristate acetate
PPARg	peroxisome proliferator activated receptor gamma
ROS	reactive oxygen species
Sirt-1	sirtuin 1
TLR4	toll-like receptor 4
TNFa	tumor necrosis factor
UM-A	urolithin metabotype A
UM-B	urolithin metabotype B
UM-0	metabotype 0
Uro	urolithin
UroA	urolithin A
UroB	urolithin B
